# Status of national health research systems in ten countries of the WHO African Region

**DOI:** 10.1186/1472-6963-6-135

**Published:** 2006-10-19

**Authors:** Joses M Kirigia, Charles Wambebe

**Affiliations:** 1 World Health Organization, Regional Office for Africa, Brazzaville, Congo; 2 Bingham University, Abuja, Nigeria; 3 International Biomedical Research in Africa, Abuja, Nigeria

## Abstract

**Background:**

The World Health Organization (WHO) Regional Committee for Africa, in 1998, passed a resolution (AFR/RC48/R4) which urged its Member States in the Region to develop national research policies and strategies and to build national health research capacities, particularly through resource allocation, training of senior officials, strengthening of research institutions and establishment of coordination mechanisms.

The purpose of this study was to take stock of some aspects of national resources for health research in the countries of the Region; identify current constraints facing national health research systems; and propose the way forward.

**Methods:**

A questionnaire was prepared and sent by pouch to all the 46 Member States in the WHO African Region through the WHO Country Representatives for facilitation and follow up. The health research focal person in each of the countries Ministry of Health (in consultation with other relevant health research bodies in the country) bore the responsibility for completing the questionnaire. The data were entered and analysed in Excel spreadsheet.

**Results:**

The key findings were as follows: the response rate was 21.7% (10/46); three countries had a health research policy; one country reported that it had a law relating to health research; two countries had a strategic health research plan; three countries reported that they had a functional national health research system (NHRS); two countries confirmed the existence of a functional national health research management forum (NHRMF); six countries had a functional ethical review committee (ERC); five countries had a scientific review committee (SRC); five countries reported the existence of health institutions with institutional review committees (IRC); two countries had a health research programme; and three countries had a national health research institute (NHRI) and a faculty of health sciences in the national university that conducted health research. Four out of the ten countries reported that they had a budget line for health research in the Ministry of Health budget document.

**Conclusion:**

Governments of countries of the African Region, with the support of development partners, private sector and civil society, urgently need to improve the research policy environment by developing health research policies, strategic plans, legislations, programmes and rolling plans with the involvement of all stakeholders, e.g., relevant sectors, research organizations, communities, industry and donors.

In a nutshell, development of high-performing national health research systems in the countries of the WHO African Region, though optional, is an imperative. It may be the only way of breaking free from the current vicious cycle of ill-health and poverty.

## Background

**Figure 1:**
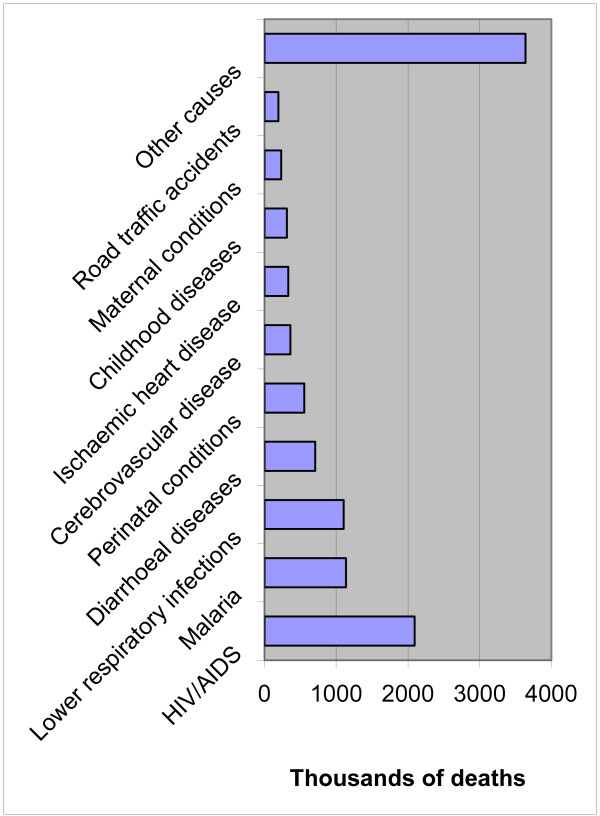
Leading causes of death in the WHO African Region. Source: WHO [1].

The people in the countries of the African Region of the World Health Organization (WHO) suffer a heavy burden of communicable and noncommunicable diseases. In 2002, 66% of the 10.7 million deaths that occurred in the Region resulted from the ten causes as shown in Figure 1[1]. HIV/AIDS, lower respiratory tract infection, malaria, diarrhoeal diseases and maternal and perinatal conditions accounted for 55% of the deaths and 54% of disability-adjusted life years (DALYs). Most of those deaths could have been averted if the people in need had access to available cost-effective interventions.

The persistent crippling burden of disease can be attributed to many causes that include: weak national and district health systems; human resources for health crisis which has been exacerbated by internal and external brain drain; 47% of the population in the Region having no access to health services, and about 50% have no access to essential drugs [2]; about 59% of pregnant women delivering babies without the assistance of skilled health personnel [3]; 64% of the population lacking sustainable access to improved sanitation facilities and 42% lacking sustainable access to an improved water source [4]; out-of-pocket expenditures constituting 51%-90% of the private health expenditure in 14 countries and 91%-100% in 24 countries [3]; 38.2% of the people in sub-Saharan Africa living below the international income poverty line of US$1 per day [5]; low investment in health development [3]; and poor governance [6].

Those challenges are compounded by weak national health research systems (NHRS), which hinder the generation of new information and knowledge for diagnosing and providing solutions; monitoring of health system performance; development and production of new technologies and health products for tackling priority diseases and health conditions; and innovating ways of accessing and putting into effective nationwide use the existing cost-effective promotive, preventive, curative, rehabilitative and care interventions.

A national health research system is a system that integrates and coordinates the vision, mission, objectives, structures, processes, cultures and outcomes of health research towards improvement in the national health system's performance of its functions of stewardship, health financing, resource creation, resource allocation and service provision and also achievement of health system goals – health, responsiveness to people's non-medical expectations and fair financial contributions [7,8].

In 1998, the WHO African Advisory Committee on Health Research and Development (AACHRD) hypothesized that the challenges confronting national health research systems included: poor environment for research; inadequate manpower; inadequate infrastructures and facilities; inaccessibility to modern technology; and lack of funds [9].

The WHO Regional Committee for Africa, in 1998, adopted a strategic health research plan for the Region [10]. It passed a resolution (AFR/RC48/R4) which urged Member States to: determine national priority research areas; draw up national research policies and strategies; build national health research capacities, particularly through resource allocation, training of senior officials, strengthening of research institutions and establishment of coordination mechanisms; develop a national health research plan; and establish a national ethics committee to ensure compliance with international ethical standards, especially regarding the conduct of clinical trials on humans [11].

The objectives of this study were to: (i) take stock of some aspects of national resources for health research in the Region; (ii) identify the current constraints facing national health research systems; and (iii) propose the way forward.

## Methods

**Figure 2:**
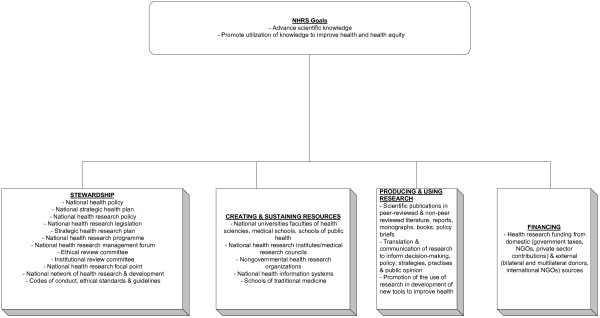
National health research systems conceptual framework. Source: Adapted from Pang et al [12].

Pang et al [12] defined a NHRS as the people, institutions, and activities whose intrinsic goals are to advance scientific knowledge and promote its utilization to improve health and health equity. The authors proposed four functions of an effective NHRS: stewardship, financing, creating and sustaining resources (health research inputs), and producing and using research. The conceptual framework for this study, which is presented in Figure 2, was adapted from Pang et al [12]. However, our study was restricted to aspects of the functions of stewardship, creating and sustaining health inputs, and financing for health research.

The primary approach used to collect data of the study reported in this paper was the questionnaire in Appendix 1 (see Additional File 1). It contained questions grouped into ten bands or categories: health research policy; health research legislation; strategic health research plan; research coordination mechanisms; health research programme; research institutes; national universities; health research financing and budget; nongovernmental organizations involved in health research; and actions needed to strengthen health research capacity.

The health research policy part had questions on: existence of an official national health policy and strategic health plan; existence of an official health research policy, including year of formulation, its contents (e.g. preamble, situation analysis of health research, vision for health research, organizational or working plan of national health research system); national health research policy statement – aims and objectives; complementarity's between national health policy and health research policy; institutions involved in its development; where it exists, whether it needs updating; where it does not exist, whether policy-makers are interested in developing it; whether support is needed from WHO in developing a health research policy; and the specific form of support needed, e.g. technical and/or financial support, guidelines, guidance for developing grant proposals, human capacity development, or sharing experiences from countries with health research policy.

The health research legislation component contained questions on: existence of a law legislating health research; whether it encapsulates ethical concerns; and the year when it was enacted.

The strategic health research plan (SHRP) component had questions on: existence of a strategic health research plan; period covered by the plan; title of the plan; whether the plan needed updating; whether WHO support was needed; and whether the plan was being implemented.

The research coordination mechanisms component asked questions related to: existence of a functional national health research system (NHRS); whether NHRS had clear terms of reference; existence of a functional national health research management forum (NHRMF); whether NHRMF had clear terms of reference; existence of a functional ethical review committee (ERC); whether ERC had written terms of reference; frequency of ERC meetings; existence of a scientific review committee (SRC); whether SRC had written terms of reference; frequency of SRC meetings; existence of health institutions with institutional review committees (IRC); existence of hospitals with ethical review committees to review clinical research proposals; existence of a national health research focal point; existence of guidelines on development of collaboration agreements on health research involving foreign institutions and agencies; and existence of a national network of health research and development which includes, among others: universities, medical research councils or institutes, representatives of nongovernmental hospitals, provincial (or regional) medical officers of health, district medical officers of health, and national medical association.

The health research programme component consisted of questions on: existence of a health research programme (HRPR); date when HRPR was constituted; whether HRPR had a mission statement; whether HRPR had clearly-defined terms of reference and an organizational structure; number of technical and support staff in HRPR; whether HRPR had a plan of action; number of computers that HRPR had; whether HRPR had e-mail and Internet connectivity; name of the government ministry or department where HRPR was housed; annual budget of the health research programme; whether HRPR undertook any research by itself; and a list of the titles of the studies undertaken by HRPR in the previous year.

The research institutes section had questions on: existence of a national health research institute (NHRI); when the institute was started; the ministry in which NHRI was housed; list of personnel in the institute (indicating their specialities), its research priorities, published research outputs for the last three years; whether NHRI had telephone facilities, fax machine(s), scanner(s); whether each research section in NHRI had a computer and a printer; whether each researcher in the NHRI had access to e-mail and Internet; existence of a memorandum of understanding (MoU) between MOH and NHRI; whether MOH commissioned NHRI to undertake operations research from time to time and a list of the studies undertaken for MOH over the last two years; whether NHRI was a WHO collaborating centre; a list of the ways in which NHRI disseminated its research; and five key enabling and constraining factors for health research in medical research councils or health research institutes.

The national universities component had questions on: list of national universities with faculties of health sciences and their contact details; whether faculties of health sciences conducted research; list of personnel (indicating their specialities), research priorities and published research for those faculties of health sciences that conducted research; number of researchers with their own computers; whether the faculties of health sciences had memoranda of understanding with MOH; whether the MoUs were for developing human resources, providing technical advice or undertaking research for MOH; and a list of five key enabling and constraining factors for health research in universities.

The health research financing and budget component had questions on: existence of a budget line for health research in the MOH budget document; the amount of money allocated by MOH to research in 2001 and 2002; the amount of MOH's overall budget in 2001 and 2002; an estimate of the total government budgetary allocation to health research in 2001; the total government budget in 2001 and 2002; the approximate amount of money from all sources spent on health research last year; how research was financed in the country, i.e. government tax revenues, private sector companies, multilateral and bilateral donor funding, local NGOs, international NGOs and others.

The section on nongovernmental organizations involved in health research had questions on: existence of NGOs in the country that undertook health research; and the names of those NGOs, their contact information and source(s) of funding.

The last section on actions needed to strengthen health research capacity asked the respondents to indicate actions that should be taken at local and international levels to stimulate health research.

The questionnaire was peer reviewed by the WHO/AFRO divisional research focal persons. However, it was not pilot-tested prior to administration. The questionnaire was developed in English and subsequently translated into French and Portuguese. Of the 46 Member States in the WHO African Region (WHO/AFRO), 21 speak French, 20 English and 5 Portuguese. It was sent by WHO diplomatic pouch, in November 2003, to each of the 46 countries through the WHO Country Representatives for facilitation and follow up.

The health research focal person in each of the countries Ministry of Health (with the support of the WHO Country Office national professional officer in charge of research) bore the responsibility for completing the questionnaire. Since the information required is not centralized at the Ministry of Health, the research focal person obtained the relevant information from the national health research institutes, national universities with faculties of health sciences, non-governmental organizations that undertake health research, etc.

The data were entered and analysed in Excel spreadsheet.

## Results

### Response rate

Only 10 (21.7%) countries (Cape Verde, Ethiopia, Equatorial Guinea, Guinea Bissau, Malawi, Mali, Mozambique, Rwanda, Senegal, and Sao Tome and Principe) out of the 46 Member States in the Region responded to the questionnaire. Therefore, the analysis reported in this paper is restricted to the ten respondent countries only. The presence or absence of various NHRS attributes per country can be found in Appendix 2 (see Additional File 2).

### National health policy, strategic health plan, health research policy, health research legislation and a strategic health research plan

Table 1 presents the availability of a national health policy (NHP), a strategic health plan (SHP) and a health research policy (HRP) in ten sub-Saharan African countries. Seven (70%) of those countries reported that they had an official NHP. Eight (80%) of them reported that they had a SHP, meaning that one of those countries did not have a NHP. Only three countries (Ethiopia, Mali and Senegal) had an official HRP. Generally, the HRP documents for the three countries that reported to have them had a preamble; a situation analysis of health research in the country; a strategic vision for health research in the country (including vision, goals, underlying values, guiding principles, research priorities, implementation strategies, resource mobilization mechanisms, and modalities for monitoring and evaluation); an organizational or working plan of the national health research system; a strategic vision for the assessment of the national health research system; and a national HRP statement – aims and objectives. Two (Ethiopia and Mali) out of the three countries that had both a NHP and a HRP reported that there were complementarities between the two documents.

Generally, countries that had a HRP reported that faculties of health sciences in national universities, medical research councils or institutes, representatives of nongovernmental hospitals, provincial (or regional) medical officers of health, district medical officers of health, national medical association(s) and administrators of HRP were involved in the process of formulating the HRP. Two (Ethiopia and Senegal) of the countries that reported to have a HRP said that it needed updating. All the seven countries that did not have a HRP reported that policy-makers were interested in developing it. Seven countries reported that WHO support was needed in the development of HRP. Those countries were asked what form of support would be needed from WHO. All of them said that they needed technical support; six countries needed guidelines on the formulation of HRP; six countries needed financial support for undertaking a health research situation analysis; all countries wanted WHO to share experiences and lessons from countries with HRP; and all countries needed support for strengthening their human capacity for implementing HRP.

Only one country (Mali) out of the ten reported to have a law relating to health research; and that country indicated that the law encapsulated ethical concerns. It is unfortunate that the other 9 (90%) countries did not have a research legislation for protecting the integrity, dignity and safety of human research subjects.

Only two (Mali and Senegal) of the ten countries reported to have a strategic health research plan. These countries indicated that their SHRP needed updating, and that they needed WHO's support for this purpose. One of the two countries reported that their plan was being implemented.

### Research coordination mechanisms

Table 2 summarizes the responses to the questions related to the existence and functioning of national research coordination mechanisms. Three countries (Ethiopia, Mali and Senegal) reported that they had a functional national health research system (NHRS) and two of these countries reported that the NHRS had clear terms of reference. Two countries (Mali and Senegal) reported the existence of a functional national health research management forum (NHRMF) with clear terms of reference.

Six (Ethiopia, Malawi, Mali, Rwanda, Mozambique and Senegal) of the ten countries had a functional ethical review committee (ERC) with written terms of reference. When the countries that reported to have an ERC were asked how regularly the committee met, two said monthly, three said quarterly, and one said the committee met whenever there were projects to review.

One-half (Ethiopia, Guinea Bissau, Mali, Senegal, and Sao Tome and Principe) of the ten countries (50%) reported to have a scientific review committee (SRC). When the countries that reported to have an SRC were asked how regularly the committee met, one said monthly, one said quarterly and three reported that it met whenever there were projects to review.

Fifty per cent of the respondent countries (Ethiopia, Malawi, Mali, Mozambique and Senegal) reported the existence of health institutions with institutional review committees (IRC). Only two countries (Ethiopia and Mozambique) reported that they had hospitals with ethics review committees to review clinical research proposals.

Eight countries (80%) reported the existence of a national health research focal point. None of the countries had national guidelines on development of collaboration agreements on health research involving health institutions and agencies outside the country.

Three countries (Mali, Mozambique and Senegal) had a national network of health research and development (NNHRD) that included universities (especially faculties of health sciences), medical research councils or institutes, provincial/regional medical officers of health, district medical officers of health and national medical associations; two of these national networks included representatives of nongovernmental hospitals as well.

### Health research programme

Table 3 shows that only two (Mali and Senegal) countries out of the ten respondent countries reported to have a health research programme. They reported that the programme had a mission statement, clearly defined the terms of reference, and had a clearly defined organizational structure and a plan of action. Three countries responded to the question, which had asked about the number of technical and support staff their health research programme had, to which they said their programme had an average of seven persons.

Three countries responded to the question asking about the number of computers owned by their programme. An average of three computers per country were reported. Two countries reported that their programme was connected to e-mail and Internet. Only one country reported that its health research programme undertook research by itself.

### Research institutes

Table 4 presents the national health research institutes' attributes. Three (Ethiopia, Mali and Mozambique) out of ten countries reported to have a national health research institute (NHRI) under Ministry of Health. All the three NHRIs were reported to have telephone facilities, a fax machine and a scanner. Two countries reported that each researcher in the NHRI had a computer and a printer and had access to e-mail and Internet.

The three countries with a NHRI reported the existence of a MoU between Ministry of Health and the institution, and that MOH commissioned the NHRI to undertake operations research from time to time. Two of the NHRIs were reported to have been designated as WHO collaborating centres. The NHRIs disseminated their research through seminars and conferences, in-house seminars, newsletters, institutional publications and international journals, and annual, quarterly and monthly reports.

Countries were asked to name five key enabling and constraining factors for health research in their medical research councils (MRC) or health research institutes (HRI). Their responses are summarized in Table 5. Research policy support was identified as the most important enabling factor in the three countries that had health research programmes. Shortage of research funding was mentioned as the most important setback to health research in MRCs and HRIs.

### National universities

Table 6 presents the health research enabling and constraining factors in national universities. Only three (Ethiopia, Mali and Senegal) out of ten countries reported the existence of a faculty of health sciences in their national university that conducted health research. They had a MoU with ministries of Health. When asked what the MoU was about, two countries indicated it was for developing human resources for health, one country said it was for technical advice, and two countries said it was for health research. The factors that facilitated national universities' health research included the existence of a school of public health; the fact that research output was a requirement for staff promotion; and the requirement for students to write a research report or dissertation in partial fulfilment of degree requirement. The constraining factors for health research in universities included deficiency in research skills among the teaching staff; inadequacy of research facilities; and lack of research grants.

### Health research financing and budget

Four (Malawi, Mali, Rwanda and Senegal) out of ten countries reported that they had a budget line for health research in the Ministry of Health budget document. Table 7 shows the sources of health research funding in order of importance. None of the countries indicated government tax revenues as a very important source for health research. Instead, multilateral and bilateral donor funding was reported to be the most important source of funding for health research. Seven countries reported the involvement of nongovernmental organizations in the provision of health resources.

### Actions needed to strengthen health research capacity

Countries were asked to indicate actions that should be taken at local and international levels to stimulate health research. Their responses are summarized in Table 8. The five most important actions needed at country level to strengthen national health research systems included: allocation of regular budget for health research and establishment of local health research financing systems; incentives for researchers and clear career development paths for health researchers; establishment of a legal framework (policy and legislation) for health research; clearly-defined structural and institutional arrangements for health research; and strengthening of human health research capacity.

The five main actions, in order of importance, needed at international level to stimulate health research capacity in African countries included: increased access to donations and funding for health research; strengthening of health research collaboration and linkages; provision of technical health research training opportunities; establishment of consultative exchange visits and forums; and promotion of networking and technical support.

## Discussion

### Key findings

The study took stock of national resources for health research in ten countries in the WHO African Region and identified constraints that faced national health research systems.

The key findings were as follows: the response rate was 21.7% (10/46); three countries had a health research policy; one country reported that it had a law relating to health research; two countries had a strategic health research plan; three countries reported to have a functional national health research system; two countries confirmed the existence of a functional national health research management forum; six countries had a functional ethical review committee; five countries had a scientific review committee; five countries reported the existence of health institutions with institutional review committees; two countries had a health research programme; and three countries had a national health research institute and a faculty of health sciences in the national university that conducted health research. Four out of ten countries reported that they had a budget line for health research in the Ministry of Health budget document.

### The way forward

The questionnaire used in this study provided a "check-list" of attributes that should be found in a functional NHRS, and the way forward re-states what was not found. Therefore, the following actions need to be taken by the ten sub-Saharan African countries to increase functionality of NHRS:

(a) All countries should have an updated national health research policy [13]. The policy should be based on a thorough situation analysis of health research in the country. It should clearly spell out the strategic vision for health research, guiding principles and underlying values, goals, research priorities, implementation framework, resource mobilization mechanisms, and modalities for monitoring and evaluation [14].

(b) All countries should ensure that the national health research policy is closely aligned with the issues, challenges and health priorities identified in the national health policy. This is necessary to ensure that the health research programme contributes to diagnosing and providing solutions to the country's public health problems.

(c) All countries should have a law governing health research. A legislation is necessary not only for governance of the national health research system and protection of intellectual property rights but, more importantly, to protect human research subjects, i.e. to ensure that international principles (e.g. beneficence, non-maleficence, autonomy, justice, dignity, truthfulness and honesty) for human experimentation are vigilantly observed [15,16].

(d) All countries should strive to develop vibrant national health research systems [13] linking people (including researchers and research users), institutions (national health research institutes or medical research councils), organizations (e.g. organizations that fund research), and mechanisms that support health research (e.g. scientific review committee, national ethics committee, institutional review committee).

(e) All countries should have an updated strategic health research plan [14,17-19] which should be translated into action through rolling annual operational plans. The strategic plan should be based on a rigorous health and health research situation analysis. It should contain a background; situation analysis (socioeconomic context; health situation; state of health services supply and demand; strengths, weaknesses, opportunities and threats); strategic health research priorities (vision, mission, goal, guiding principles, objectives, targets, strategic thrusts, expected results/outcomes, activities and performance indicators); resource requirements, including human resources, building space, vehicles, equipment, materials and supplies, information, communication and technology (ICT); finance plan (containing prospective estimates cost, available funds, financing gap and ways of bridging the gap); implementation framework specifying the roles and responsibilities of various people, institutions and organizations involved in health research; monitoring and evaluation, including mechanisms, schedule and cost; conclusion; and appendices.

(f) All countries should have a health research programme, which would ensure that: (i) the strategic health research plan is translated into action through rolling annual operational plans; (ii) monitoring and evaluation indicators are developed [20]; (iii) monitoring and evaluation [21] of the implementation of the strategic health research plan is done and the results fed back into the people, institutions and organizations involved in health research; (iv) there is coordination between various stakeholders; (v) the south-south and north-south health research partnerships are created or strengthened if they already exist; (vi) a national database of health research is established and regularly updated; (vii) national health research grants are carefully administered; (viii) the capacity of national health systems decision-makers (at headquarters, regions, districts and health facilities) for accessing and utilizing research findings is strengthened; (ix) the growth of national health research journals is supported; and (x) the ethical review systems are developed at all levels of the health system to vigilantly assure the safety and dignity of human research subjects [22].

(g) Invest in computer, e-mail and Internet connectivity across the national health research system at all levels (headquarters, regions, districts and health facilities) of the national health system [23]. This will facilitate access to the internationally available published health research materials. It will also provide a cost-effective avenue for disseminating research undertaken within the country through the online peer reviewed journals.

(h) All countries should design a compensation system for health researchers that would attract, motivate and retain them [24]. Compensation refers not only to extrinsic rewards such as monetary rewards (e.g. salary, bonus, commission, pay incentive) and benefits (e.g. life insurance, health insurance, retirement package, paid holidays, paid public holidays, food services, recreation) but also intrinsic rewards (e.g. achieving personal goals, autonomy, recognition, promotion opportunities, working conditions, intellectually stimulating work). The objectives of an equitable compensation system are to attract good applicants, retain good employees, motivate employees and comply with government human resource management legislation [25,26]. The compensation for people working in tertiary institutions/bodies (e.g. medical schools, schools of public health, nursing schools, national health research institutes, medical research councils) should be directly related to accurately evaluated/appraised high performance.

(i) All countries should "invest at least 2% of national health expenditures in research and research capacity strengthening, and at least 5% of project and programme aid for the health sector from development aid agencies should be earmarked for research and research capacity strengthening" [27]. The 58^th ^World Health Assembly resolution WHA58.34 [13] urged Member States to implement the above-mentioned recommendation made by the Commission on Health Research for Development.

(j) All countries are encouraged to critically review the recommendations and action plan contained in the World Report on Knowledge for Better Health with a view to implementing them [28].

### Limitations of this study

The study reported in this paper had a number of limitations. Firstly, we did not make any attempt to evaluate the extent to which NHRS achieved their intrinsic goals of advancement of scientific knowledge and promotion of its utilization to improve health and health equity [12,20]. Thus, our study is a partial study of NHRS.

Secondly, this study was restricted to the NHRS functions of stewardship, financing and creating and sustaining resources or health research inputs. The questionnaire did not have any questions on the production and use of health research outputs. In addition, it did not assess the availability of non-ICT equipment used in health research (e.g. laboratories and reagents), functioning motor vehicles for fieldwork, office space, supportive supervision, human resources for health research motivation and retention, among others. This information was missed partly due to our ignorance full range of the functions of NHRS at the time of developing the questionnaire and also because the questionnaire was not pilot-tested prior to administration.

Thirdly, the health research focal person in each of the countries Ministry of Health bore the responsibility for completing the questionnaire in consultation with the national health research institutes, national universities with faculties of health sciences, non-governmental organizations that undertake health research, etc. We do not know how many people were interviewed in each of those organizations/institutions in the process of completing the questionnaire. Thus, it is not clear how representative the views of the persons interviewed were of the entire NHRS in each country. In order to increase validity, future NHRS studies in the African Region should consider drawing lessons from the approaches employed in the Council on Health Research for Development (COHRED) – and the World Health Organization Regional Office for South-East Asia (SEARO) – sponsored case studies on national health research. Those case studies constituted groups of individuals with different perspectives or contributions to national health research activities and capacities, collectively discussed and responded to the questionnaire [29,30]. D'Souza and Sadana [29] and Sadana et al [30] reviewed the existing case studies and found that there was need for refinement and better documentation of methods used to develop case studies.

Fourthly, only 10 out of 46 countries in the Region responded. Thus, whereas the results reported in this paper may be representative of the 10 countries that responded, they cannot be generalized for the 46 WHO Member States in the African Region. In preparation for the second global ministerial conference on Research for Health in 2008, the World health Organization in collaboration with COHRED, UNICEF/UNDP/World Bank/WHO Special Programme for Research and Training in Tropical Diseases (TDR) and health research networks in the Region among others, are planning to undertake an in-depth comprehensive assessment of national health research, health information and knowledge systems in the African Region. The assessment will describe the stewardship, financing, research inputs and outputs, dissemination and impact of NHRS; describe the resources, indicators, data sources, data management, information products, dissemination and use of health information; describe how knowledge from research, health information and other sources is created, captured, disseminated, applied and used for improving, protecting, or restoring health; based on the findings of the mapping, develop national and regional strategic plans for advocacy, resource mobilization and implementation [31]. The proposed assessment intends to map the full range of stakeholders (sectors, organizations/institutions, disciplines, networks and users) in different stages of research, information and knowledge generation, synthesis and use within each country and across the region.

A number of actions may enable the new proposed assessment in all the 46 countries to achieve a higher response rate than that reported in this paper: (i) the WHO management should hold individual WHO Country Representatives fully accountable for country-level follow-up to ensure completion of the data collection instrument(s); (ii) the WHO management should give written assurance to Member States that the outcomes of the study would help WHO and partners to tailor their technical and financial support to the specific country needs; (iii) the WHO management should apprise the countries that the results from the study will constitute Africa's contribution to the 2008 Global Ministerial Conference on Research for Health; (iv) WHO Country Representatives should negotiate with the relevant national authorities to constitute multi-stakeholder research for health steering committees to oversee the data collection, preliminary analysis and dissemination (through country-based workshops); (v) use remunerated national professional researchers to administer the data collection instrument(s); (vi) organize workshops at the regional economic communities headquarters to train the researchers on the purpose of the proposed study, data collection instruments, field methodology, and potential usefulness of the research outcomes in the development of NHRS, health management information systems, and knowledge management systems; (vii) involve the African Health Research Forum in undertaking the study.

Fifthly, the respondent countries did not provide estimates of the total government allocation to research. Thus, it was not possible to know whether countries were making concerted efforts to achieve the target for investing at least 2% of national health expenditures in research and research capacity strengthening, as recommended by the Commission on Health Research for Development [27].

## Conclusion

Evidence is critically needed to guide strengthening of national health systems to facilitate scale-up of proven interventions and health services needed for the achievement of national health development goals and the internationally agreed goals, including the Millennium Development Goals (MDGs) [32,33]. We cannot have the evidence that the national health policy-makers need to formulate appropriate health policies and take relevant action to strengthen national health systems without NHRS that adequately perform their functions of stewardship, creating and sustaining health research resources, producing and using research results, and health research financing [12,20,29,30].

Unfortunately, out of the ten countries included in this study, seven had no health research policy; nine had no law relating to health research; eight had no strategic health research plan; eight had no functional national health research management forum; four had no functional ethical review committee; five had no scientific review committee; five had no institutional review committees; eight had no health research programme; and six countries reported no budget line for health research in the Ministry of Health budget document.

Governments of countries of the African Region, with the support of development partners, private sector and civil society, urgently need to improve the research policy environment by developing health research policies, strategic plans, legislations, programmes and rolling plans with the involvement of all stakeholders, e.g., relevant sectors, research organizations, communities, industry and donors.

In a nutshell, development of high-performing national health research systems in the countries of the WHO African Region, though optional, is an imperative. It may be the only way of breaking free from the current vicious cycle of ill-health and poverty.

## Abbreviations

AACHRD – African Advisory Committee on Health Research and Development

COHRED – Council on Health Research for Development

DALYs – disability-adjusted life years

ERC – ethical review committee

HRP – health research policy

HRPR – health research programme

IRC – institutional review committee

MOH – Ministry of Health

MOU – memorandum of understanding

MRC – medical research councils

NGO – nongovernmental organization

NNHRD – national network of health research and development

NHP – national health policy

NHRI – national health research institute

NHRMF – national health research management forum

NHRS – national health research system

SEARO – World Health Organization Regional Office for South-East Asia

SHP – strategic health plan

SHRP – strategic health research plan

SRC – scientific review committee

TDR – UNICEF/UNDP/World Bank/WHO Special Programme for Research and Training in Tropical Diseases

WHO – World Health Organization

WHO/AFRO – World Health Organization Regional Office for Africa

## Competing interests

The author(s) declare that they have no competing interests.

## Authors' contributions

JMK and CW participated equally in the design, analysis and interpretation of data, and writing of the manuscript.

## Pre-publication history

The pre-publication history for this paper can be accessed here:



## Supplementary Material

Additional file 1 Appendix 1: Questionnaire on Country Resources for Health Research. This is the questionnaire that was used to collect data on the status of health research – policy, legislation, research plan, coordination mechanisms, programme, institutes, and national universities – in the ten sub-Saharan Africa countries. Click here for file

Additional file 2 Appendix 2: National health research systems profiles for ten sub-Saharan Africa countries. The data provided represent the detailed status of health research policy, legislation, research plan, coordination mechanisms, programme, institutes, and national universities in the study countries. Click here for file

## Figures and Tables

**Table 1:** Availability of a national health policy, strategic health plan and health research policy

**Health research policy**	No. of countries ***n *= 10**
Official national health policy (NHP)	7
Strategic health plan (SHP)	8
Official health research policy (HRP)	3
HRP with a preamble	3
HRP with health research situation analysis	3
HRP with a strategic vision for health research	3
Working plan for national health research system (NHRS)	2
Strategic vision for assessment of NHRS	2
National health research policy statement (aims, objectives)	2
Complementarity's between NHP and HRP	2
Universities involved in formulation of HRP	3
Medical research councils	3
Representatives of nongovernmental hospitals	2
Provincial or regional medical officers of health	3
District medical officers of health	2
National medical association	3
Administrators of HRP	3
HRP needs updating	2
HRP does not exist but policy-makers interested in developing it	7
WHO support needed in development of HRP	7
Form of support: technical	7
Guidelines on formulation of HRP	6
Financial support for health research situation analysis	6
Technical guidance in development of grant proposals	6
Human capacity development for HRP implementation	7
Sharing of experiences and lessons from countries with HRP	7

**Table 2:** Existence and functioning of national research coordination mechanisms

**Research coordination mechanisms**	**Number of countries *n *= 10**
Existence of a functional NHRS	3
NHRS has clear terms of reference	2
Existence of a functional national health research management forum (NHRMF)	2
NHRMF has clear terms of reference	2
Existence of a functional ethical review committee (ERC)	6
ERC has written terms of reference	6
Scientific review committee (SRC) exists	4
SRC has written terms of reference	3
Existence of health institutions with institutional review committees (IRC)	5
Existence of hospitals with ERCs to review clinical research proposals	2
Existence of a national health research focal point	8
Existence of national guidelines on development of collaboration agreements on health research involving health institutions and agencies outside the country	0
Existence of a national network of health research and development (NNHRD) that includes universities	3
Existence of a NNHRD that includes medical research councils or institutes	3
Existence of a NNHRD that includes representatives of nongovernmental hospitals	2
Existence of a NNHRD that includes provincial/regional medical officers of health	3
Existence of a NNHRD that includes district medical officers of health	3
Existence of a NNHRD that includes national medical association	3

**Table 3:** Presence of a health research programme

**Health research programme-related information**	**Number of countries *n *= 10**
Has a health research programme (HRPR)	2
Programme has mission statement	2
Programme has clearly defined terms of reference	2
Programme has a clearly defined organizational structure	2
Programme has a plan of action	2
Average number of computers in a programme	3
Programme is connected to e-mail and Internet	2
Programme is housed in Ministry of Health	2
Programme undertakes research itself	1

**Table 4:** National health research institutes attributes

**Research institutes information**	**Number of countries *n *= 10**
Has a national health research institute (NHRI)	3
NHRI is under Ministry of Health	3
NHRI has telephone facilities	3
NHRI has fax machines	3
NHRI has scanners	3
Each researcher in NHRI has a computer and a printer	2
Each researcher in NHRI has access to e-mail and Internet	3
Existence of MoU between MoH and NHRI	3
MoH from time to time commissions NHRI to undertake operations research	3
NHRI is a WHO collaborating centre	2
NHRI disseminates research through seminars and conferences	3
NHRI disseminates research through in-house seminars	3
NHRI disseminates research through newsletters, institutional publications and international journals	3
NHRI disseminates research through annual, quarterly and monthly reports	3

**Table 5:** Enabling and constraining factors for health research in medical research councils or health research institutes

**Enabling factors**	**Number of countries *n *= 10**
Requirement of research for graduation	2
Proclamation and institutional support	1
Availability of small grants	2
Internationally recognized	1
Research policy support	3
Political support	2
Existence of research coordination & evaluation mechanism	1
***Constraining factors***	
Shortage of funding	3
Lack of network for dissemination	2
Deficiency in research capacity/skills	2
Lack of health research networking at national and international levels	2
Lack of incentives for researchers & deficiency of health research facilities	2
Lack of utilization of research results	1

**Table 6:** National universities health research enabling and constraining factors

**National universities**	**Number of countries *n *= 10**
Faculties of health sciences conduct research	3
Faculties of health sciences with a MoU with MoH	3
MoU is about developing human resources for MoH	2
MoU is about technical advice to MoH	1
MoU is for undertaking research for MoH	2
***Enabling factors for health research in universities***	
Research consideration in staff promotion	2
Research is a requirement for students	2
Existence of a public health school	1
***Constraining factors for health research in universities***	
Research skills deficiency	2
Research grants lacking	3
Inadequacy of research facilities	1

**Table 7:** Importance of various sources of health research funding

**Sources of funding for health research**	**Rating by countries on a scale of 1 (very important) to 6 (least important)**					
	
	***1***	***2***	***3***	***4***	***5***	***6***
Government tax revenues	0	0	3	0	2	1
Private sector companies	2	0	0	2	1	0
Multilateral and bilateral donor funding	3	1	0	0	1	1
Local NGOs	0	1	3	1	1	0
International NGOs	1	2	0	1	0	1

**Table 8:** Actions needed to strengthen health research capacity

**Actions needed at country level to stimulate health research capacity**	**Number of countries *n *= 10**
Incentives for researchers and clear career development paths for researchers	6
Establish networking with institutions & individual researchers	1
Allow tax exemptions for research inputs	1
Allocate regular budget for health research & establish local financing systems	8
Establish legal framework (policy & legislation) for health research	5
Clearly define structural and institutional arrangements for health research	4
Establish alternative mechanisms of research dissemination	1
Strengthen human health research capacity	4
Increase awareness on the need for national health research agenda	1
Equip research institutions	2
Encourage utilization of research findings in decision-making	1
**Actions needed at international level to stimulate health research capacity**	
Strengthen health research collaboration and linkages	5
Provide technical training opportunities	4
Establish more access to donations and funding	8
Establish consultative exchange visits and forums	4
Promote networking and technical support	4
Strengthen health research systems	2
Help with health research equipment	2
Nominate a research focal point at WHO country office	1
