# Supplementary material for: Status of national health research systems in ten countries of the WHO African Region
Source: BMC Health Serv Res. 2006 Oct 19;6:135. doi: 10.1186/1472-6963-6-135 (PMC1622748; doi:10.1186/1472-6963-6-135)
Supplement: Additional file 2 — Appendix 2: National health research systems profiles for ten sub-Saharan Africa countries. The data provided represent the detailed status of health research policy, legislation, research plan, coordination mechanisms, programme, institutes, and national universities in the study countries. [file 1472-6963-6-135-S2.doc]

**Appendix 2: National health research systems profiles for ten sub-Saharan Africa countries**

| **Health Research Policy** | | | | | | | | | | | |
| --- | --- | --- | --- | --- | --- | --- | --- | --- | --- | --- | --- |
| **Yes=1; No=0** | **Ethiopia** | **Malawi** | **Sao Tome & Principe** | **Mali** | **Cape Verde** | **Rwanda** | **Guinea**  **Bissau** | **Equatorial**  **Guinea** | **Mozambique** | **Senegal** | **TOTALS** |
| Official national health policy (NHP) | 1 | 1 | 1 | 1 | 1 | 0 | 1 | 0 | 1 | 0 | 7 |
| Strategic health plan (SHP) | 1 | 0 | 1 | 1 | 1 | 0 | 1 | 1 | 1 | 1 | 8 |
| Official health research policy (HRP) | 1 | 0 | 0 | 1 | 0 | 0 | 0 | 0 | 0 | 1 | 3 |
| HRP with a preamble | 1 | 0 | 0 | 1 | 0 | 0 | 0 | 0 | 0 | 1 | 3 |
| HRP with health research situation analysis | 1 | 0 | 0 | 1 | 0 | 0 | 0 | 0 | 0 | 1 | 3 |
| HRP with a strategic vision for health research | 1 | 0 | 0 | 1 | 0 | 0 | 0 | 0 | 0 | 1 | 3 |
| Working plan for the NHRS | 0 | 0 | 0 | 1 | 0 | 0 | 0 | 0 | 0 | 1 | 2 |
| Strategic vision for assessment of NHRS | 0 | 0 | 0 | 1 | 0 | 0 | 0 | 0 | 0 | 1 | 2 |
| National health research policy statement (aim, objectives) | 1 | 0 | 0 | 1 | 0 | 0 | 0 | 0 | 0 | 0 | 2 |
| Complementarity’s between NHP and HRP | 1 | 0 | 0 | 1 | 0 | 0 | 0 | 0 | 0 | 0 | 2 |
| Universities involved in formulation of HRP | 1 | 0 | 0 | 1 | 0 | 0 | 0 | 0 | 0 | 1 | 3 |
| Medical research councils involved in formulation of HRP | 1 | 0 | 0 | 1 | 0 | 0 | 0 | 0 | 0 | 1 | 3 |
| Representatives of non-governmental hospitals involved in formulation of HRP | 0 | 0 | 0 | 1 | 0 | 0 | 0 | 0 | 0 | 1 | 2 |
| Provincial or regional medical officers of health involved in formulation of HRP | 1 | 0 | 0 | 1 | 0 | 0 | 0 | 0 | 0 | 1 | 3 |
| District medical officers of health involved in formulation of HRP | 0 | 0 | 0 | 1 | 0 | 0 | 0 | 0 | 0 | 1 | 2 |
| National medical association involved in formulation of HRP | 1 | 0 | 0 | 1 | 0 | 0 | 0 | 0 | 0 | 1 | 3 |
| Administrators of HRP involved in formulation of HRP | 1 | 0 | 0 | 1 | 0 | 0 | 0 | 0 | 0 | 1 | 3 |
| HRP needs updating | 1 | 0 | 0 | 0 | 0 | 0 | 0 | 0 | 0 | 1 | 2 |
| HRP does not exist but policy-makers interested in developing it | 0 | 1 | 1 | 0 | 1 | 1 | 1 | 1 | 1 | 0 | 7 |
| WHO support needed in revision/development of HRP | 0 | 1 | 1 | 1 | 1 | 1 | 1 | 1 | 1 | 0 | 8 |
| Technical support needed from WHO | 1 | 1 | 1 | 0 | 1 | 1 | 1 | 1 | 0 | 0 | 7 |
| Guidelines on formulation of HRP needed | 0 | 1 | 0 | 0 | 1 | 1 | 1 | 1 | 1 | 0 | 6 |
| Financial support for health research situation analysis needed from WHO | 1 | 1 | 0 | 0 | 0 | 1 | 1 | 1 | 1 | 0 | 6 |
| WHO technical guidance needed in development of grant proposals | 1 | 1 | 0 | 1 | 0 | 1 | 1 | 0 | 1 | 0 | 6 |
| Need for human capacity development for HRP implementation | 1 | 1 | 0 | 1 | 1 | 1 | 1 | 1 | 1 | 0 | 8 |
| Need for sharing of experiences and lessons from countries with HRP | 1 | 1 | 0 | 1 | 1 | 1 | 1 | 0 | 1 | 0 | 7 |
| **Health Research Legislation** | | | | | | | | | | | |
| Has a law relating to health research | 0 | 0 | 0 | 1 | 0 | 0 | 0 | 0 | 0 | 0 | 1 |
| Law includes ethical concerns | 0 | 0 | 0 | 1 | 0 | 0 | 0 | 0 | 0 | 0 | 1 |
| **Strategic Health Research Plan** | | | | | | | | | | | |
| Has a strategic health research plan (SHRP) | 0 | 0 | 0 | 1 | 0 | 0 | 0 | 0 | 0 | 1 | 2 |
| SHRP needs updating | 0 | 0 | 0 | 1 | 0 | 0 | 0 | 0 | 0 | 1 | 2 |
| WHO support needed in updating SHRP | 0 | 0 | 0 | 1 | 0 | 0 | 0 | 0 | 0 | 1 | 2 |
| SHRP is being implemented | 0 | 0 | 0 | 0 | 0 | 0 | 0 | 0 | 0 | 1 | 1 |
| **Research Coordination Mechanisms** | | | | | | | | | | | |
| Existence of a functional NHRS | 1 | 0 | 0 | 1 | 0 | 0 | 0 | 0 | 0 | 1 | 3 |
| NHRS have clear terms of reference | 0 | 0 | 0 | 1 | 0 | 0 | 0 | 0 | 0 | 1 | 2 |
| Existence of a functional national health research management forum (NHRMF) | 0 | 0 | 0 | 1 | 0 | 0 | 0 | 0 | 0 | 1 | 2 |
| NHRMF has clear terms of reference | 0 | 0 | 0 | 1 | 0 | 0 | 0 | 0 | 0 | 1 | 2 |
| Existence of a functional ethical review committee (ERC) | 1 | 1 | 0 | 1 | 0 | 1 | 0 | 0 | 1 | 1 | 6 |
| ERC have written terms of reference | 1 | 1 | 0 | 1 | 0 | 1 | 0 | 0 | 1 | 1 | 6 |
| Have a scientific review committee (SRC) | 1 | 0 | 1 | 1 | 0 | 0 | 1 | 0 | 0 | 1 | 5 |
| SRC have written terms of reference | 0 | 0 | 1 | 1 | 0 | 0 | 0 | 0 | 0 | 1 | 3 |
| Existence of health institutions with institutional review committees (IRC) | 1 | 1 | 0 | 1 | 0 | 0 | 0 | 0 | 1 | 1 | 5 |
| Existence of hospitals with ERCs to review clinical research proposals | 1 | 0 | 0 | 0 | 0 | 0 | 0 | 0 | 1 | 0 | 2 |
| Existence of a national health research focal point | 1 | 1 | 1 | 1 | 0 | 1 | 1 | 0 | 1 | 1 | 8 |
| Existence of national guidelines on development of collaboration agreements on health research involving health institutions and agencies outside the country | 0 | 0 | 0 | 0 | 0 | 0 | 0 | 0 | 0 | 0 | 0 |
| Existence of a national network of health research and development (NNHRD) that includes universities | 0 | 0 | 0 | 1 | 0 | 0 | 0 | 0 | 1 | 1 | 3 |
| Existence of a NNHRD that includes medical research councils or institutes | 0 | 0 | 0 | 1 | 0 | 0 | 0 | 0 | 1 | 1 | 3 |
| Existence of a NNHRD that includes representatives of non-governmental hospitals | 0 | 0 | 0 | 1 | 0 | 0 | 0 | 0 | 0 | 1 | 2 |
| Existence of a NNHRD that includes provincial/regional medical officers of health | 0 | 0 | 0 | 1 | 0 | 0 | 0 | 0 | 1 | 1 | 3 |
| Existence of a NNHRD that includes district medical officers of health | 0 | 0 | 0 | 1 | 0 | 0 | 0 | 0 | 1 | 1 | 3 |
| Existence of a NNHRD that includes national medical associations | 0 | 0 | 0 | 1 | 0 | 0 | 0 | 0 | 1 | 1 | 3 |
| **Health Research Programme** | | | | | | | | | | | |
| Has a health research programme (HRPR) | 0 | 0 | 0 | 1 | 0 | 0 | 0 | 0 | 0 | 1 | 2 |
| Programme has mission statement | 0 | 0 | 0 | 1 | 0 | 0 | 0 | 0 | 0 | 1 | 2 |
| Programme has clearly defined terms of reference | 0 | 0 | 0 | 1 | 0 | 0 | 0 | 0 | 0 | 1 | 2 |
| Programme have a clearly defined organizational structure | 0 | 0 | 0 | 1 | 0 | 0 | 0 | 0 | 0 | 1 | 2 |
| Average number of technical and support staff in a programme | 0 | 0 | 0 | 10 | 0 | 0 | 0 | 0 | 1 | 4 | 7.5 |
| Programme have a plan of action | 0 | 0 | 0 | 1 | 0 | 0 | 0 | 0 | 0 | 1 | 2 |
| Average number of computers in a programme | 0 | 0 | 0 | 3 | 0 | 0 | 0 | 0 | 1 | 2 | 6 |
| Programme is connected to e-mail and internet | 0 | 0 | 0 | 1 | 0 | 0 | 0 | 0 | 1 | 1 | 3 |
| Programme is housed in the Ministry of Health | 0 | 0 | 0 | 1 | 0 | 0 | 0 | 0 | 1 | 1 | 3 |
| Programme undertakes research itself | 0 | 0 | 0 | 0 | 0 | 0 | 0 | 0 | 0 | 1 | 1 |
| **Research Institutes** | | | | | | | | | | | |
| Have a national health research institute (NHRI) | 1 | 0 | 0 | 1 | 0 | 0 | 0 | 0 | 1 | 0 | 3 |
| NHRI is under the Ministry of Health | 1 | 0 | 0 | 1 | 0 | 0 | 1 | 0 | 1 | 0 | 4 |
| NHRI has telephone facilities | 1 | 0 | 0 | 0 | 0 | 0 | 1 | 0 | 1 | 0 | 3 |
| NHRI has fax machines | 1 | 0 | 0 | 0 | 0 | 0 | 1 | 0 | 1 | 0 | 3 |
| NHRI has scanners | 1 | 0 | 0 | 0 | 0 | 0 | 1 | 0 | 1 | 0 | 3 |
| Each researcher in the NHRI has a computer and a printer | 0 | 0 | 0 | 0 | 0 | 0 | 1 | 0 | 1 | 0 | 2 |
| Each researcher in the NHRI have access to e-mail and internet | 1 | 0 | 0 | 1 | 0 | 0 | 1 | 0 | 1 | 0 | 4 |
| Existence of memorandum of understanding between MoH and NHRI | 1 | 0 | 0 | 1 | 0 | 0 | 1 | 0 | 0 | 0 | 3 |
| MoH from time to time commissions NHRI to undertake operations research | 1 | 0 | 0 | 1 | 0 | 0 | 0 | 0 | 1 | 0 | 3 |
| The NHRI is a WHO collaborating centre | 1 | 0 | 0 | 1 | 0 | 0 | 0 | 0 | 0 | 0 | 2 |
| NHRI disseminates research through seminars & conferences | 1 | 0 | 0 | 1 | 0 | 0 | 1 | 0 | 1 | 0 | 4 |
| NHRI disseminates research through in-house seminars | 1 | 0 | 0 | 0 | 0 | 0 | 1 | 0 | 1 | 0 | 3 |
| NHRI disseminates research through news letters, institutional publications & international journals | 1 | 0 | 0 | 1 | 0 | 0 | 1 | 0 | 1 | 0 | 4 |
| NHRI disseminates research through annual, quarterly & monthly reports | 1 | 0 | 0 | 0 | 0 | 0 | 1 | 0 | 1 | 0 | 3 |
| ***Enabling factors for health research in MRCs or HRIs*** | | | | | | | | | | | |
| Requirement of research for graduation | 1 |  | 0 | 0 | 0 | 0 | 0 | 0 | 1 | 0 | 2 |
| Proclamation and institutional support | 1 |  | 0 | 0 | 0 | 0 | 0 | 0 | 0 | 0 | 1 |
| Availability of small grants | 1 |  | 0 | 0 | 0 | 0 | 1 | 0 | 0 | 0 | 2 |
| Internationally recognized | 1 |  | 0 | 0 | 0 | 0 | 0 | 0 | 0 | 0 | 1 |
| Research policy support | 1 |  | 0 | 1 | 0 | 0 | 1 | 0 | 0 | 0 | 3 |
| Research policy | 0 |  | 0 | 1 | 0 | 0 | 1 | 0 | 0 | 0 | 2 |
| Research coordination & evaluation mechanism | 0 |  | 0 | 1 | 0 | 0 | 0 | 0 | 0 | 0 | 1 |
| ***Constraining factors for health research in MRCs or HRIs*** | | | | | | | | | | | |
| Shortage of funding | 1 | 0 | 0 | 1 | 0 | 0 | 1 | 0 | 1 | 0 | 4 |
| Lack of network for dissemination | 1 | 0 | 0 | 1 | 0 | 0 | 0 | 0 | 0 | 0 | 2 |
| Deficiency in research capacity/skills | 1 | 0 | 0 | 0 | 0 | 0 | 1 | 0 | 0 | 0 | 2 |
| Lack of funds and networking | 1 | 0 | 0 | 0 | 0 | 0 | 1 | 0 | 0 | 0 | 2 |
| lack of incentives and deficiency of facilities | 1 | 0 | 0 | 0 | 0 | 0 | 0 | 0 | 1 | 0 | 2 |
| Lack of utilization of research results | 0 | 0 | 0 | 1 | 0 | 0 | 0 | 0 | 0 | 0 | 1 |
| **National Universities** | | | | | | | | | | | |
| Faculties of health sciences conduct research |  | 1 | 0 | 0 | 0 | 1 | 0 | 0 | 1 | 1 | 4 |
| Faculties of health sciences with a MOU with MOH | 1 | 0 | 0 | 1 | 0 | 0 | 0 | 0 | 0 | 1 | 3 |
| MOU is about developing human resources for the MoH | 0 | 0 | 0 | 1 | 0 | 0 | 0 | 0 | 1 | 0 | 2 |
| MoU is about technical advise to the MOH | 0 | 0 | 0 | 0 | 0 | 0 | 0 | 0 | 1 | 0 | 1 |
| MoU is for undertaking research for the MOH | 0 | 0 | 0 | 1 | 0 | 0 | 0 | 0 | 1 | 0 | 2 |
| ***Enabling factors for health research in Universities*** | | | | | | | | | | | |
| Research consideration in staff promotion | 1 | 0 | 0 | 0 | 0 | 0 | 0 | 0 | 1 | 0 | 2 |
| Research is a requirement for students | 1 | 0 | 0 | 0 | 0 | 0 | 0 | 0 | 1 | 0 | 2 |
| Existence of a public health school | 0 | 0 | 0 | 0 | 0 | 1 | 0 | 0 | 0 | 0 | 1 |
| ***Constraining factors for health research in Universities*** | | | | | | | | | | | |
| Research skills deficiency | 1 | 0 | 0 | 0 | 0 | 1 | 0 | 0 | 0 | 0 | 2 |
| Research grants lacking | 1 | 0 | 0 | 0 | 0 | 0 | 0 | 0 | 0 | 0 | 1 |
| Research facilities deficient | 1 | 0 | 0 | 0 | 0 | 0 | 0 | 0 | 0 | 0 | 1 |
| Deficiency in funding | 1 | 0 | 0 | 0 | 0 | 1 | 0 | 0 | 0 | 0 | 2 |
| **Health Research Financing and Budget** | | | | | | | | | | | |
| Existence of a budget line for health research in the MoH budget document | 0 | 1 | 0 | 1 | 0 | 1 | 0 | 0 | 0 | 1 | 4 |
|  |  |  |  |  |  |  |  |  |  |  |  |
| ***Importance of various sources of health research funding the country (Rating by countries on a scale of '1=very important' to '6=least important')*** | | | | | | | | | | | |
| Government tax revenues | 3 | 5 |  | 3 |  | 5 |  |  | 6 |  |  |
| Private sector companies | 5 | 4 |  | 1 |  | 4 |  |  | 1 |  |  |
| Multi-lateral and bi-lateral donor funding | 1 | 2 | 1 | 6 |  | 1 |  |  | 5 |  |  |
| Local NGOs | 4 | 3 |  | 2 |  | 3 |  |  | 3 |  |  |
| International NGOs | 2 | 1 |  | 4 |  | 2 |  |  | 6 |  |  |
| **Existence of Non-Governmental Organizations Involved in Health Research** | | | | | | | | | | | |
| 1=NGO involved in health research exists; 0=Does not exist | 1 | 1 | 0 | 1 | 0 | 0 | 1 | 1 | 1 | 1 | 7 |
| **Actions Needed to Strengthen Health Research Capacity** | | | | | | | | | | | |
| ***Actions needed at country level to stimulate health research capacity*** | | | | | | | | | | | |
| Incentives for researchers and clear career paths | 1 | 0 | 0 | 1 | 0 | 1 | 1 | 0 | 1 | 1 | 6 |
| Establish networking with institutions & individual researchers | 1 | 0 | 0 | 0 | 0 | 0 | 0 | 0 | 0 | 0 | 1 |
| Allow tax exemptions for research inputs | 1 | 0 | 0 | 0 | 0 | 0 | 0 | 0 | 0 | 0 | 1 |
| Allocate regular budget for health research | 1 | 1 | 0 | 1 | 1 | 1 | 1 | 0 | 1 | 0 | 7 |
| Establish legal framework (policy & legislation) for health research | 1 | 0 | 0 | 0 | 1 | 1 | 1 | 0 | 1 | 0 | 5 |
| Clearly define structural and institutional arrangements for health research | 1 | 0 | 0 | 1 | 1 | 0 | 1 | 0 | 0 | 0 | 4 |
| Establish alternative mechanisms of research dissemination | 1 | 0 | 0 | 0 | 0 | 0 | 0 | 0 | 0 | 0 | 1 |
| Establish local financing systems | 1 | 0 | 0 | 0 | 0 | 0 | 0 | 0 | 0 | 0 | 1 |
| Strengthen research capacity | 0 | 1 | 0 | 1 | 1 | 1 | 0 | 0 | 0 | 0 | 4 |
| Increase awareness on the need for national health research agenda | 0 | 1 | 0 | 0 | 0 | 0 | 0 | 0 | 0 | 0 | 1 |
| Equip research institutions | 0 | 0 | 0 | 1 | 0 | 0 | 0 | 0 | 0 | 1 | 2 |
| Utilization of research findings | 0 | 0 | 0 | 1 | 0 | 0 | 0 | 0 | 0 | 0 | 1 |
| ***Actions needed at international level to stimulate health research capacity*** | | | | | | | | | | | |
| Strengthen health research collaboration and linkages | 1 | 0 | 0 | 1 | 0 | 1 | 0 | 0 | 1 | 1 | 5 |
| Provide technical training opportunities | 1 | 0 | 0 | 1 | 1 | 1 | 0 | 0 | 0 | 0 | 4 |
| Establish more access to donations and funding | 1 | 1 | 0 | 1 | 1 | 1 | 1 | 0 | 1 | 1 | 8 |
| Establish consultative exchange visits and forum | 1 | 1 | 0 | 0 | 0 | 1 | 0 | 0 | 0 | 1 | 4 |
| Promote networking and technical support | 1 | 0 | 0 | 0 | 0 | 0 | 1 | 0 | 1 | 1 | 4 |
| Strengthen health research systems | 1 | 0 | 0 | 0 | 0 | 0 | 1 | 0 | 0 | 0 | 2 |
| Help with health research equipment | 1 | 0 | 0 | 1 | 0 | 0 | 0 | 0 | 0 | 0 | 2 |
| Nominate a research focal point at WHO country office | 0 | 0 | 0 | 0 | 0 | 1 | 0 | 0 | 0 | 0 | 1 |

Notes: 1=affirmative (presence of an attribute) and 0=absence of the attribute in question
